# Adult intussusception: a challenge to laparoscopic surgery?

**DOI:** 10.7717/peerj.14495

**Published:** 2022-11-30

**Authors:** Mingze Sun, Zhongmin Li, Zhenbo Shu, Qi Wu, Xue Liu

**Affiliations:** 1 Department of General Surgery, Tongde Hospital of Zhejiang Province, Hangzhou, Zhejiang, China; 2 Department of Gastrointestinal Colorectal and Anal Surgery, China-Japan Union Hospital of Jilin University, Changchun, Jilin, China; 3 Obstetrics and Gynacology, Tongde Hospital of Zhejiang Province, Hangzhou, Zhejiang, China

**Keywords:** Adult intussusception, Laparoscopic surgery, Computed tomography (CT)

## Abstract

**Background:**

Intussusception can occur at any age and is common in children but less common in adults. This study aimed to evaluate our experience of 51 adult intussusception and study the etiology, clinical manifestations, diagnosis, and treatment.

**Methods:**

This analysis assessed the clinical manifestations, etiology, diagnosis, and treatment of adult intussusception in 51 adult patients at the Department of Gastrointestinal Surgery of China-Japan Union Hospital of Jilin University from January 2010 to December 2020.

**Results:**

The mean age of the cohort was 54.43 ± 18.21 years, and 42 patients were diagnosed by abdominal ultrasonography and abdominal computed tomography (CT). Among them, 76.5% (39/51) had abdominal pain, 11.8% (6/51) had blood in stool, and 5.9% (3/51) had a palpable abdominal mass. Of these, 62.7% had tumors: malignant accounted for 39.2% (20/51) and benign accounted for 23.5% (12/51). CT is the preferred imaging method with a sensitivity of 92.2%, while colonoscopy provides a complementary diagnosis in patients involving the colon. All patients underwent surgical treatment, including 21.6% (11/51) laparoscopic surgery, 74.5% (38/51) open surgery, and 5.9% (3/51) intussusception reduction during the operation. The average operation time of the open group was 133.27 ± 43.75 min and the average hospital stay was 16.24 ± 12.55 days, while the average operation time of the laparoscopic group was 140.50 ± 46.15 mins, and the average hospital stay was 16.60 ± 16.98 days (*P* > 0.05).

**Conclusion:**

Adult intussusception is a rare disease in clinic. Laparoscopic surgery can be useful and safe for adult intussusception.

## Introduction

Intussusception is a condition in which a section of the intestine is inserted into a distal neighbor and causes obstruction of the passage of intestinal contents. It is common in children but uncommon in adults (Agha, 1986). The adult intussusception accounts for only 5% of total cases (Gueye et al., 2018) and causes 1% to 5% of intestinal obstructions (Kim et al., 2018). Adult intussusception is a rare surgical emergency that often leads to misdiagnosis or missed diagnosis.

The clinical manifestations of adult intussusception are often nonspecific, it can be acute paroxysmal colic or chronic, dull pain that lasts for months or longer (Balogun et al., 2019; Farhan et al., 2020). As the disease progresses, symptoms of intestinal obstruction such as abdominal distention and vomiting may occur, and in severe cases, intestinal necrosis may occur, which can cause fever, loss of consciousness, and even death. Due to the atypical clinical manifestations of adult intussusception, it is difficult to diagnose before surgery, therefore, we need to use adjuvant testing. This article will discuss which of the various auxiliary tests works best.

Adult intussusception usually requires surgical treatment (Yakan et al., 2009; Ikram et al., 2018). Although laparoscopic equipment and surgical techniques have recently been improved and applied to adult intussusception, there is no consensus on safety and efficacy. The optimal management for adult intussusception is still controversial. This study was done to review and document the etiology, clinical manifestations, diagnosis, and treatment of adult intussusception during the past 11 years, so as to make some contributions to the diagnosis and treatment of adult intussusception.

## Materials and Methods

This study was conducted in the Department of Gastrointestinal Surgery of China-Japan Union Hospital of Jilin University (Changchun, China). The study was approved by the ethics committee of Tongde Hospital of Zhejiang Province. We have got a waiver of informed consent in the ethical approval. Ethical examination batch number Zhejiang Tongde fast review character no. (2021)072. The medical records of adult intussusception patients treated from January 2010 to December 2020 were reviewed. All patients underwent abdominal ultrasound, plain abdominal radiography, CT scan, and colonoscopy before surgery to confirm the diagnosis. Data on patients’ age, sex, clinical manifestations, diagnosis-assisting tests, clinicopathological types, and surgical treatment were collected and analyzed. Patients with intussusception were diagnosed by abdominal ultrasound, plain abdominal film, CT, colonoscopy, or surgical exploration. Patients receiving laparoscopic-assisted surgery were compared to those receiving open surgery, and the operative time, hospital stay, and postoperative complications were compared. Parameters, such as clinical manifestations, assistive examination, clinicopathological type, and surgical treatment, were analyzed. All open surgeries were performed by surgeons skilled in gastrointestinal surgery, while laparoscopic surgery was performed by surgeons with expertise in laparoscopy.

### Inclusion criteria

(1) Intussusception was definitely diagnosed; (2) preoperative assessment of patients who could tolerate laparoscopic or open surgery.

### Exclusion criteria

(1) Organ dysfunction can not tolerate surgery and anesthesia. (*e.g.*, cardiopulmonary dysfunction, *etc*.); (2) Intraoperative exploration was combined with other operations; (3) Coagulation is abnormal: (*e.g.*, thrombin/prothrombin time exceeded the normal control by more than 3 s, fibrinogen is below 2 g, *etc*.); (4) Follow-up and communication disorders in patients.

### localization and duration

Adult intussusception was classified into acute (duration ≤3 days), subacute (4–14 days), and chronic (>14 days) according to the duration of symptoms (Balogun et al., 2019). Adult intussusception was classified according to its location in the gastrointestinal tract: (1) small intestine type: intussusception (limited to the small intestine); (2) ileocolonic type: ileocecal intussusception or ileocecal intussusception, including invagination of the ileum through the ileocecal valve; (3) colonic type: intussusception involving the colon; (4) sigmoid-rectal type: intussusception involving the sigmoid colon and rectum (Lianos et al., 2013).

### Statistical methods

IBM® SPSS® statistics software version 26.0 (IBM Corp., Armonk, NY, USA) was used for data analysis. Categorical variables were described as frequencies and percentages. Continuous variables were expressed as mean ( $\bar x$) ± standard deviation (SD). Continuous variables were reported as means ± SDs (if the distribution was normal) or medians with ranges (if the distribution was skewed). For statistical analysis, Student’s t test was used to compare means of numerical variables between the two groups. *P* < 0.05 indicated a statistically significant difference between the two groups.

## Results

In the current study, 51 patients aged 17–92 years were diagnosed with adult intussusception. There were 21 males with an average age of 57.50 ± 15.88 years (28–86 years) and 30 females with an average age of 52.30 ± 19.55 years (17–92 years). The male-female ratio was 1:1.4, *P* = 0.323 (Table 1). There were 12 cases (23.5%) of small intestine type, 27 cases (52.9%) of ileocolonic type, six cases (11.8%) of colonic type, and six cases (11.8%) of sigmoid-rectal type (Table 2).

**Table 1:** Demographics in patients.

	Male	Female	T	*P*	Sex ratio
N	21	30			0.7
Age	57.50 ± 15.88	52.30 ± 19.55	0.999	0.323	

**Table 2:** Clinical characteristics in patients.

	*N*	Percentage (%)
Clinical manifestations		
Abdominal pain	39	76.5
Bloody stools	6	11.8
Abdominal mass	3	5.9
Nausea and vomiting	30	55.8
Weight loss	8	15.7
Ileus	30	58.8
Course of disease		
Acute	20	39.2
Subacute	19	37.3
Chronic	12	23.5
Pathological types		
Tumor	32	67.2
Malignant	20	39.2
Benign	12	23.5
Intestinal inflammatory disease	13	25.5
Idiopathic	3	5.9
Adhesion of small intestine	1	2.0
Peutz-Jeghers syndrome	2	3.9
Types		
Small intestine type	12	23.5
Ileocolonic type	27	52.5
Colonic type	6	11.8
Sigmoid-rectal type	6	11.8

The pathological examinations of 51 patients of intussusception showed that tumors accounted for 62.7% (32/51): malignant tumors accounted for 39.2% (20/51), benign tumors accounted for 23.5% (12/51). In addition, 25.5% (13/51) of patients could be attributed to Intestinal inflammatory disease, 3.9% (2/51)of patients had small intestinal Peutz-Jeghers syndrome, 5.9%(3/51) of patients were Idiopathic, and 2% (1/51) was caused by intestinal adhesions (Table 2).

Out of the 51 patients, 76.5% (39/51) had abdominal pain, 11.8% (6/51) had bloody stools, 5.9% (3/51) had palpable abdominal mass, 55.8% (30/51) had nausea and vomiting, and 15.7% (8/51) had weight loss. Moreover, 58.8% (30/51) of patients had different degrees of ileus, with 39.2% (20/51) presenting acute symptoms, 37.3% (19/51) had subacute symptoms, and 23.5% (12/51) exhibiting chronic symptoms (Table 2).

About the diagnostic modalities of the patients, the plain abdominal film was performed on 15 patients, 0 was diagnosed with intussusception (Sensitivity 0.0%). Abdominal plain film is mainly used for the diagnosis of intestinal obstruction, which showed typical hydro-air levels, nevertheless there are no typical signs for intussusception. Abdominal ultrasound was performed on 15 patients; among them, eight were diagnosed with intussusception (Sensitivity 53.3%). A total of 27 patients underwent abdominal CT scans, of which 25 were diagnosed with intussusception (Sensitivity 92.2%); seven patients with adult intussusception underwent colonoscopy, and the cause was identified (Table 3). The diagnoses were performed transoperatively.

**Table 3:** Diagnostic radiological evaluation.

Variable	Subjects, *n* (%)	Sensitivity, *n* (%)
Abdominal plain film	15 (29.4)	0 (0)
Abdominal CT	27 (52.9)	25 (92.6)
Abdominal ultrasound	15 (29.4)	8 (53.3)
Colonoscopy	7 (5.8)	7 (100)

**Note:**

CT, computed tomography.

All patients underwent surgery, including one case of small bowel reduction due to intussusception caused by intraoperative adhesions, two patients of reduction due to rectal intussusception, and one case of Hartmann surgery due to malignant rectal lesions, and the remaining patients underwent intussusception and reduction and resection. Among them, there were 40 patients of open surgery: three of bowel reduction, nine of partial small bowel resection, eight of ileocecal resection, 14 of right hemicolectomy, two of left hemicolectomy, one of partial resection of transverse colon, one case of Hartmann surgery, one of rectotomy, and 11 of laparoscopic surgery (two patients of laparoscopic partial resection of the small intestine, five patients of laparoscopic ileocecal resection, three patients of Laparoscopic right hemicolectomy, and one case of laparoscopic rectal resection). The average duration of surgery in the open group was 133.27 ± 43.75 min, the average length of hospital stay was 16.24 ± 12.55 days, the average operation time of the laparoscopic group was 140.50 ± 46.15 min, and the average length of hospital stay was 16.60 ± 16.98 days. The average operation time of the open group was less than that of the laparoscopic group, albeit not significantly (*P >* 0.05). In the open group, there were three patients of postoperative incision infection, one of pulmonary infection, two of lower limb venous embolism, and one of abdominal effusion, while no postoperative complications were detected in the laparoscopic group (Table 4).

**Table 4:** Statistics of adult intussusception surgical treatment.

	OG (*n* = 40)	LG (*n* = 11)	*P*
Operation methods			
Bowel reduction	3	0	
Small bowel resection	9	2	
Ileocecal resection	8	5	
Right hemicolectomy	14	3	
Transverse colectomy	1	0	
Rectectomy	1	1	
Hartmann surgery	1	0	
Left hemicolectomy	1	0	
Sigmoidectomy	2	0	
Length of hospital stay (mean ± SD)	16.24 ± 12.55	16.60 ± 16.98	0.937
Duration of surgery (mean ± SD)	133.27 ± 43.75	140.50 ± 46.15	0.649
Postoperative complications			
Incision infection	3	0	1.000
pulmonary infection	1	0	1.000
Lower limb venous embolism	2	0	1.000
Abdominal effusion	1	0	1.000

**Note:**

OG, Open group means patients who done the open surgery; LG, laparoscopic group means patients who done the laparoscopic surgery; SD, standard deviation For variables that are less than 5, we used the Fisher exact test.

## Discussion

Intussusception is considered a rare condition in adults. The variability of the clinical manifestations makes diagnosis difficult. Surgical resection of intestinal lesions is the main treatment for adult intussusception laparoscopic surgery is emerging as an alternative to open surgery; however, it is controversial in the treatment of adult intussusception.

Briggs et al. (2014) reported that adult intussusception patients are more common in males (male: female ratio 2.9:1) with an average onset age of 40 years. The data showed that there were more females than males, the ratio of males to females was 0.7 (1:1.4), and *P* > 0.05 indicated no statistical significance. We agree with Eisen, Cunningham & Aufses (1999) that adult intussusception was not related to the gender of patients. Presently, pathological changes and inflammatory stimuli in the intestinal lumen can change intestinal peristalsis, leading to intussusception (Marinis et al., 2009). The small intestine type of intussusception accounts for the majority of the adult patients (Rea et al., 2007). However, in our study, the ileocolonic type is the most common, followed by the small intestine type, which was similar to the report by Goh et al. (2006). Intussusception can also be classified by etiology or root cause: idiopathic, benign, or malignant (Hong et al., 2019). So, the most common intussusception of adult intussusception is the ileocolonic type, and benign or malignant tumors are the most common causes of adult intussusception.

The clinical manifestations of adult intussusception vary and are usually occult and intermittent rather than acute (Balogun et al., 2019). Symptoms include abdominal pain, bloating, nausea and vomiting, changes in bowel habits, and bleeding in the lower gastrointestinal tract. Abdominal pain is considered the most common symptom, occurring in 70–100% of patients, when malignant tumors cause intussusception, symptoms include weight loss, black stools, or a palpable abdominal mass (Marinis et al., 2009). In our study, symptoms were usually acute, subacute and chronic, with varying duration. Abdominal pain was the most common symptom, followed by nausea and vomiting. Other non-specific symptoms included bloody stools, abdominal mass, and weight loss. The symptoms of intussusception are recurrent and self-limiting, which makes the diagnosis difficult. Symptoms suggestive of malignancy such as bloody stools and weight loss were found in the minority of patients in this study. Some studies reported that the preoperative diagnosis rate was <50% (Gupta et al., 2011b).

**Figure 1:**
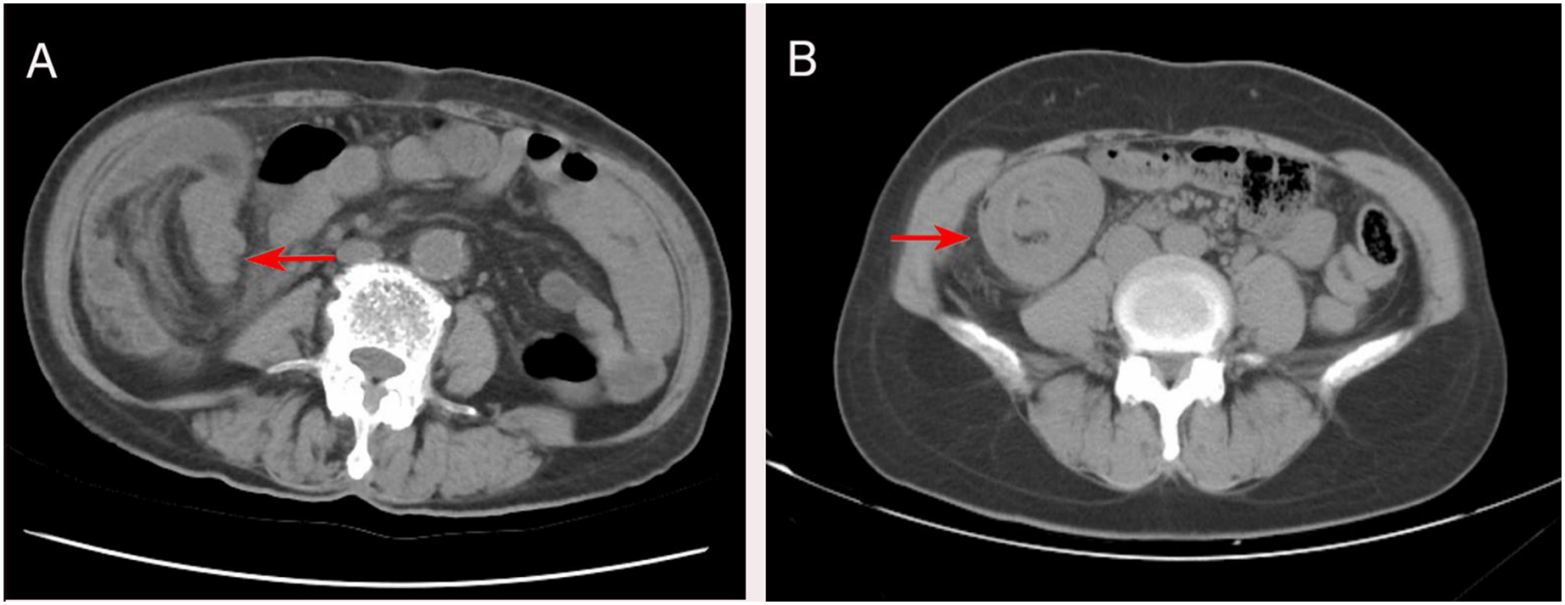
(A) CT at the level of the abdomen shows ileocecal intussusception (arrow). (B) CT at the level of the abdomen shows a round, target-shaped mass in the right abdomen (arrow).

The preoperative diagnosis of intestinal intussusception in adults is often difficult. The abdominal plain film shows dilated bowel loops and fluid-gas planes, like the features of intestinal obstruction, but has no specific sign for intussusception. Plain abdominal films are useful when an obstruction is suspected (Marinis et al., 2009). In this study, plain abdominal films were used in 15 patients, which could not be identified as intussusception and had specific limitations. On abdominal ultrasonography, intussusception showed a concentric circle sign and target ring syndrome, which is useful in evaluating the intussusception in adults presenting a palpable abdominal mass, with a >90% accuracy rate (Wang et al., 2009). In this study, 15 patients were examined by abdominal ultrasound, with a diagnostic rate of 53.5%. Ultrasound is helpful to improve the diagnosis of suspected intussusception. It is a helpful tool especially in children; it can be useful in adults when an abdominal mass can be palpated but may be limited in the presence of loop air. On the other hand, a colonoscopy may be a useful diagnostic tool for patients with subacute or chronic intermittent ileus (Rahimi et al., 2016). It is the most useful method for adult intussusception involving the colon, terminal ileum, and cecum that confirms intussusception, location, and biopsy with respect to the diagnosis and the planning of surgery (Shenoy, 2017; Elena et al., 2012). In this study, seven patients with adult intussusception (confirmed mass) underwent colonoscopy, while the procedure should be avoided in patients with acute obstruction as it may increase the risk of perforation (El-Sergany et al., 2015). We agree with the Gupta et al. (2011a) report that colonoscopy is most accurate in ileocolic and colonic adult intussusception. The CT findings of intussusception were target sign, concentric circle sign, and pseudorenal sign (Fig. 1). CT displays the length and diameter of intussusception, the three-dimensional view of the intestine and surrounding organs, the starting point, type, and position of intussusception, mesenteric vascular system, the possibility of strangulation, and the possibility of partial or complete intestinal obstruction (Gore et al., 2015). Currently, CT is the most sensitive scan for diagnosing intussusception in adults, with a diagnostic accuracy of 58–100% (Honjo et al., 2015). In addition, CT can determine the most appropriate treatment method and avoid unnecessary surgery (Rafailidis et al., 2017). In the current study, 27 patients underwent CT, with a diagnostic rate of 92.2%. CT scan can determine the severity of adult intussusception and the best treatment, thereby providing strong evidence for the diagnosis of intussusception. With significant advancements in CT technology, CT may help clinicians not only to differentiate intussusception from other abdominal emergencies, but also to avoid unnecessary surgery. In this study, 27 patients underwent abdominal CT scans and intussusception was diagnosed in 25 patients, with CT being the most sensitive diagnostic modality. CT has proven to be a useful test for evaluating these patients. Also, colonoscopy is recommended to supplement the diagnose of intussusception.

Currently, surgical treatment is the primary treatment for most adult intussusception patients (Akbulut, 2012). However, the optimal surgical approach remains controversial because the main reason for the original recommendation of whole intussusception resection was the theoretical risk of venous embolization of tumor cells during intestinal canal operation and the risk of penetrating the ischemic, fragile, and edematous intestinal canal, which might lead to the implantation of tumor cells and microorganisms into the peritoneal cavity (Shenoy, 2017; Kang et al., 2020). In recent years, due to the progress of laparoscopic equipment and surgical technology, as well as its advantages of rapid recovery, less pain, and minimal scarring (Llorente et al., 2018), laparoscopic surgery for adult intussusception is being increasingly performed and has been reported to be feasible for adult intussusception (Ruiz-Tovar et al., 2013). Laparoscopic exploration is effective for the diagnosis of adult intussusception, avoiding unnecessary incisions. However, there is no consensus on the safety and effectiveness of laparoscopic surgery for adult intussusception. Tartaglia et al. (2014) reported that laparoscopic surgery for adult intussusception is a safe and feasible treatment, especially when the preoperative diagnosis is unclear (Shimazaki et al., 2015).

In this study, we did not find any significant differences in the duration of surgery and the incidence of postoperative complications between the laparoscopic and open groups (*P* > 0.05). Postoperative incision infection was the most common postoperative complication in the open group. The laparoscopic group had fewer complications than the open group. It can be seen that the common complication of open surgery is incision infection, which may be caused by intestinal wall edema, malnutrition, or acute indications caused by intestinal obstruction. Laparoscopic surgery has smaller incisions, less tissue manipulation and less tissue damage, thus reducing the incidence of infection complications. Siow et al. (2020) showed that segmentectomy for intussusception in adults cannot be removed because most of the lesions may have pathological causes, and conservative treatment may be ineffective. Due to the different causes and locations of intussusception, there is currently no standard laparoscopic surgery. During laparoscopic exploration, once the location of intussusception is found, it can be excised or eviscerated and processed externally using a small incision. A biopsy may be performed when malignant lesions are suspected. This study indicated that surgery is the predominant treatment for intussusception in adults. In some cases, laparoscopy is a useful adjunct to open surgical techniques (Kang & Gu, 2020). Although laparotomy or laparoscopic enterectomy is the ideal method, cases using laparoscopic techniques have no postoperative complications. First, the most common cause of ileocolonic intussusception is primary adenocarcinoma. Considering the high incidence rate of primary adenocarcinoma, colonic intussusception should be removed without reduction to avoid potential intraluminal implantation or venous tumor dissemination. Second, the incidence of primary malignancy in small intestinal intussusception is lower than in colon intussusception, and inappropriate reduction attempts can lead to intestinal wall tears and peritoneal contamination. We recommend removing the intussusception without attempting to reduce it. In non-acute ileocolonic intussusception, preoperative colonoscopy is helpful to distinguish benign and malignant lesions before surgery, develop appropriate surgical methods, and avoid excessive bowel resection. Finally, we agree with the Zubaidi, Al-Saif & Silverman (2006) report that small-bowel intussusception should be reduced before resection if the underlying etiology is suspected to be benign or if the resection required without reduction is deemed to be massive. Large bowel should generally be resected without reduction because pathology is mostly malignant. For experienced physicians, laparoscopic treatment of intussusception in adults is safe and feasible. However, we recommend caution to patients with acute obstruction of flatulence, as the visibility of flatulence may be poor and intestinal manipulation may further increase the risk of perforation and the incidence of surgery (Siow & Mahendran, 2014). The current study was a retrospective design with a small sample size; moreover, since this study is a single-center study with a single study population, there was the possibility of selection and recommendation bias.

## Conclusion

In conclusion, adult intussusception is a rare disease in clinical practice. Due to non-specific clinical manifestations, preoperative diagnosis is difficult, and it is easy to miss diagnosis or misdiagnosis. Preoperative CT is helpful for the diagnosis and evaluation of intussusception, and in non-acute ileocolonic intussusception, preoperative colonoscopy is helpful to distinguish benign and malignant lesions before surgery, develop appropriate surgical methods, and avoid excessive bowel resection. In the current era with advances in laparoscopic techniques, laparoscopic treatment of intussusception in adults is safe and feasible, and is no longer a challenge. In the future, we will conduct a retrospective analysis with a larger sample size by multi-hospital combination to validate some of our inferences.

## Supplemental Information

10.7717/peerj.14495/supp-1 Supplemental Information 1 Raw data.

## Additional Information and Declarations

### Data Availability

The following information was supplied regarding data availability: The raw measurements are available in the Supplemental File.
